# A Systematic Review and Meta-Analysis of Premenstrual Syndrome with Special Emphasis on Herbal Medicine and Nutritional Supplements

**DOI:** 10.3390/ph15111371

**Published:** 2022-11-08

**Authors:** Arshiya Sultana, Md Belal Bin Heyat, Khaleequr Rahman, Radhika Kunnavil, Mohamed Joonus Aynul Fazmiya, Faijan Akhtar, Sumbul, Juan Luis Vidal Mazón, Carmen Lili Rodríguez, Isabel De La Torre Díez

**Affiliations:** 1 Department of Ilmul Qabalat wa Amraze Niswan, National Institute of Unani Medicine, Ministry of AYUSH, Bengaluru 560091, Karnataka, India; 2 IoT Research Center, College of Computer Science and Software Engineering, Shenzhen University, Shenzhen 518060, China; 3 Centre for VLSI and Embedded System Technologies, International Institute of Information Technology, Hyderabad 500032, Telangana, India; 4 Department of Science and Engineering, Novel Global Community Educational Foundation, Hebersham, NSW 2770, Australia; 5 Department of Ilmul Saidla, National Institute of Unani Medicine, Ministry of AYUSH, Bengaluru 560091, Karnataka, India; 6 Department of Biostatistics, National Institute of Unani Medicine, Ministry of AYUSH, Bengaluru 560091, Karnataka, India; 7 School of Computer Science and Engineering, University of Electronic Science and Technology of China, Chengdu 611731, China; 8 Higher Polytechnic School, Universidad Europea Del Atlántico, Isabel Torres 21, 39011 Santander, Spain; 9 Department of Project Management, Universidad Internacional Iberoamericana, Campeche 24560, Mexico; 10 Faculty of Engineering, Universidade Internacional do Cuanza, Estrada Nacional 250, Bairro Kalua-Panda, Cuito-Bié 250, Angola; 11 Department of Signal Theory and Communications and Telematic Engineering, University of Valladolid, Paseo de Belén 15, 47011 Valladolid, Spain

**Keywords:** dietary supplements, female health, herbal medicine, PMS, PRISMA, reproductive health, unani medicine, Web of Science, software

## Abstract

Herbal medicine and nutritional supplements are suggested to treat premenstrual somatic and psycho-behavioural symptoms in clinical guidelines; nonetheless, this is at present based on poor-quality trial evidence. Hence, we aimed to design a systematic review and meta-analysis for their effectiveness in alleviating premenstrual symptoms. The published randomized controlled trials (RCTs) were extracted from Google scholar, PubMed, Scopus and PROSPERO databases. The risk of bias in randomized trials was assessed by Cochrane risk-of-bias tool. The main outcome parameters were analysed separately based on the Premenstrual Symptom Screening Tool and PMTS and DRSP scores. Secondary parameters of somatic, psychological, and behavioural subscale symptoms of PSST were also analysed. Data synthesis was performed assuming a random-effects model, and standardized mean difference (SMDs) was analysed using SPSS version 28.0.0 (IBM, Armonk, NY, USA). A total of 754 articles were screened, and 15 RCTs were included (*n* = 1211 patients). Primary results for participants randomized to an intervention reported reduced PSST (*n* = 9), PMTS (*n* = 2), and DSR (*n* = 4) scores with (SMD = −1.44; 95% CI: −1.72 to −1.17), (SMD = −1.69; 95% CI: −3.80 to 0.42) and (SMD = 2.86; 95% CI: 1.02 to 4.69) verses comparator with substantial heterogeneity. Physical (SMD = −1.61; 95% CI = −2.56 to −0.66), behavioural (SMD = −0.60; 95% CI = −1.55 to 0.35) and mood (SMD = 0.57; 95% CI = −0.96 to 2.11) subscale symptom groupings of PSST displayed similar findings. Fifty-three studies (*n* = 8) were considered at low risk of bias with high quality. Mild adverse events were reported by four RCTs. Based on the existing evidence, herbal medicine and nutritional supplements may be effective and safe for PMS.

## 1. Introduction

Premenstrual somatic and psycho-behavioural symptoms are categorized by a cyclic pattern that is obvious within the luteal phase [1], and they significantly affect quality of life with more frequent visits to hospitals parenting problems, decreased work productivity, and impaired individual and societal relationships [2,3,4]. It is a common and recurrent gynaecological problem [1], and 20–30% of reproductive-age women experience clinically significant premenstrual syndrome (PMS) symptoms [5]. The main psychological range of PMS symptoms includes anxiety and depression [3,6].

The aetiology of PMS is unclear; hence, definitive treatment is not available. However, several possible treatments have been established based on symptomatic relief [1,6]. Despite a rational success rate of pharmacotherapies (GnRH agonists OCP, selective serotonin reuptake inhibitors benzodiazepines) and non-pharmacological management (aerobic exercise, cognitive behavioural therapy, reduction in caffeine intake, education, and increased calcium and carbohydrate intake) for PMS, these therapies have considerable side effects [5,7,8]. Women may also prefer and are inclined towards complementary and alternative treatment/therapies (CAMS), including nutritional supplements and herbal medicines over medical or surgical treatment [9,10,11]. Various Unani medicinal plants [12] have been expected to lessen PMS symptoms, as they are beneficial natural substitutes [6]. RCTs on Unani medicinal plants such as *Crocus sativus* Linn (saffron/zafran), *Borage officinalis* Linn (borage/gouzaban), *Vitex agnus castus* Linn (chaste berry/shambalu), *Matricaria chamomilla* Linn (chamomile/baboona), *Zingiber officinale* Roscoe (ginger/zanjabeel), *Foeniuclum vulgare* Mill (fennel/saunf), and *Pimpinella anisum* Linn (anise seed/anisoon) were investigated for PMS symptoms [13]. These herbs and nutritional supplements are proven to have anti-depressant, anti-anxiety, sedative, SSRIs inhibitor and GABA A receptor effects together with antioxidant, anti-inflammatory and analgesic properties [14].

Earlier, systematic reviews were conducted on PMS including herbal medicine and acupuncture [15], including *M. chamomilla* [6], and *V. agnus castus* [16]. Canning et al. [17] explored herbs and dietary supplements for PMS. Pearce et al. (2020) conducted a systematic review and meta-analysis on exercises for PMS. Research [18] explored the efficacy of *Rosa damascena* Mill (rose/gul surkh) on anxiety and other symptoms. Csupor et al. [19], for the management of PMS, compared the efficacy of BNO 1095 and Ze 440 to placebo. The researchers systematically reviewed and included 18 RCTs of Iranian herbal medicines and their effectiveness and safety for PMS [20]. Tu et al. [21] suggested that communication with parents and education probably reduce parental stress and anxiety. However, a current update on the systematic review and meta-analysis of RCTs on herbs and nutritional supplements lessening premenstrual somatic and psycho-behavioural symptoms as per the modified Cochrane risk-assessment tool was not accessible as per our information. Therefore, we explored RCTs for systematic review, meta-analysis and risk of bias assessment as per the modified Cochrane risk assessment tool to provide objective data for the efficacy and safety of herbs and nutritional supplements in relieving premenstrual somatic and its psycho-behavioural symptoms. Additionally, we summarized plant metabolites and their mechanism of action in selected RCTs.

### 1.1. Study Aim and Research Question

This research is proposed to determine the safety and efficacy of herbs and nutritional supplements on premenstrual somatic and psycho-behavioural symptoms. An up-to-date systematic review and meta-analysis of RCTs that compare herbal medicine and nutritional supplements to placebo/conventional medical treatment were conducted. We examined the effect of herbal medicine/nutritional supplements on overall premenstrual symptoms, as well as the three symptoms’ domains: somatic, psychological and behavioural.

Hence, this paper explains the following research questions (RQs):

(i) What is the role of a systematic review with risk assessment on PMS?
(ii) How to design the meta-analysis of RCTs based on high-quality studies related to PMS with herbal medicine and nutritional supplements?
(iii) What is the comprehensive presentation of the mechanism of action in plant metabolites and bioactive molecules?
(iv) How to design a database based on network visualization, world cloud, and previously published articles?
(v) What is the main research gap and what is the future in the area of PMS regarding herbal medicine and nutritional supplements?

### 1.2. Main Contributions of This Study

Our key contributions here are:

(i) To design an up-to-date systematic review and meta-analysis of RCTs to determine the efficacy and safety of herbal medicines and nutritional supplements with their mechanism of action on premenstrual somatic and psycho-behavioural symptoms.
(ii) To determine the risk of bias in randomized controlled trials.
(iii) To design a database such as the number of authors, university/institution, research area-wise and country-wise on previously published publications.
(iv) To design a comprehensive picture based on previous studies and present a study using network visualization and word cloud.
(v) To explore the research breaches and prospects.

### 1.3. Paper Structure

This study is organized as follows: a systematic review, risk of bias assessment and meta-analysis using PRISMA with Consort statement [22], world clouds [23], network visualization based on keywords, discussion including major findings, comparison with previous literature, mechanism of action, strength, research gap, implication of research in clinical practices, and conclusions.

## 2. Methods

A complete method was thoroughly organized with the collection of data and steps of analysis, including PRISMA guidelines for RCTs [22,23,24,25,26] per the checklist [27]. The protocol was registered at PROSPERO, University of New York (CRD42022344752, dated 17 July 2022).

The following steps were performed in the methods: (a) planning and developing a protocol, (b) registry of the protocol in PROSPERO as per PRISMA-P for the protocol [28] and PRISMA checklist for publication, (c) performed a complete literature search for information source and search strategies, (d) data extraction (selection, data management and collection process), (e) outcomes, (f) risk bias and quality assessment as per the Cochrane risk assessment guidelines, (g) strategy for data analyses and measures of effect, (h) analysis of subgroups or subsets, and (i) reporting results [28,29,30].

### 2.1. Eligibility Criteria, Study Selection, and Participants

The articles were selected for eligibility in two steps. After a thorough review to avoid bias, two researchers independently analysed the data, which were extracted from the screened articles, and the data were organized. In the first step, the articles were screened based on keywords, titles and abstracts. Published and unpublished RCTs from indexed journals with considerable reliability were included. The duplicate publications were deleted. The inclusion criteria were participants of reproductive age reporting regular menstrual cycle (21–35 days) with premenstrual somatic and psycho-behavioural symptoms diagnosed by DSM, DSRP, ACOG or other diagnostic scales using either herbal medicine and/or nutritional supplements in RCTs. At least two cycles of intervention were given per oral. As for the control interventions, a patient who did not receive any treatment as a placebo control, or those who accepted simple Western medicine as a control intervention were included. Furthermore, clinical trials where at least 8 weeks of intervention were administered were included. Full-text access to English language articles was included to validate the description of an article more specifically. There was no limitation to whether they study was published or not. Additionally, there were no restrictions on citizenship, nationality, region, and source of cases. Patients with severe systemic diseases, known psychiatric patients, pregnancy or lactation were excluded. We also excluded other routes of administration of the medicine. The experiments were limited to humans. Non-RCTs, quasi-RCTs, series of case reports, conference papers, posters, editorials, and unreliable data were excluded. In addition, the botanical name of the plants was verified and spelled according to the source World Flora Online (http://www.worldfloraonline.org, accessed on 9 October 2022).

### 2.2. Information Data Source and Search Strategies

We explored online databases (Google Scholar, PubMed, Scopus, and PROSPERO) to collect data from 2008 to 2022, focusing on randomized controlled trials on premenstrual somatic and psycho-behavioural symptoms among women. The MeSH keywords, title and abstract explored for the literature search were: “Premenstrual Dysphoric Disorders”, “Premenstrual Syndrome”, “Nutritional supplements and premenstrual syndrome”, “Herbal medicine and premenstrual syndrome”, and “psycho-behavioural and premenstrual syndrome”. Rayyan online software (https://www.rayyan.ai, accessed on 3 August 2022) was used to identify duplication, randomized controlled trials and exclusion from the publication.

### 2.3. Data Extraction

To determine eligibility and relevance, we explored the titles, abstracts, and keywords of all articles to be included in this paper. Then, a systematic examination of the full articles was performed. The final decisions on inclusion were made, and articles were selected from 2008 to 2022. Four researchers independently analysed and extracted the data. For any disagreement, a fifth researcher reviewed the data for the confirmation. The PRISMA and Consort Statement for the randomized controlled trial checklist was referred for assessment. Detailed records regarding authors, sample size, participants, research design, tools for data collection, randomization allocation, blinding, intervention type, duration of intervention, outcome, and adverse effects were documented. Extraneous studies and inadequate quantitative data were excluded. Studies that provided post-treatment data were estimated as per meta-analysis guidelines.

### 2.4. Outcomes

The main outcome parameters were analysed separately based on premenstrual symptom screening tool (PSST), premenstrual tension score (PMTS) and daily severity reporting of symptom overall scores. Secondary parameters of somatic, psychological, and behavioural subscale symptoms of PSST were also analysed. Adverse events/side effects/withdrawals for adverse effects were also reviewed. Additional outcomes were adverse events and cure rate.

### 2.5. Risk of Bias (RoB) and Quality Assessment (QA)

Any inconsistency in the data was fixed by agreement. We evaluated the methodological quality using a modified Cochrane Risk of Bias tool for RCTs. Bias was measured based on “high, low, or unclear for individual elements from five domains, i.e., selection, reporting, performance, detection and attrition bias”.

### 2.6. Statistical Methods

Statistical methods were performed for the strategy of data synthesis, the measure of effect, meta-bias (es) and confidence in cumulative evidence. Assuming that the true effect sizes differed between studies as a result of study-related characteristics, the average weighted effect sizes were estimated using a random-effects model (REM). Standardized mean difference (SMD) and 95% confidence interval (CI) were used to summarize continuous outcomes. The effect size was determined by dividing the mean pre-post value difference by the SD into the experimental and control groups. To determine whether the variance between studies was greater than the variance within studies, a Q test statistic (chi-square distributed) was computed. The Q test and I^2^ statistics were used to evaluate heterogeneity. I^2^ values of 25%, 50%, and 75% were regarded as mild, moderate, and high, heterogeneity, respectively. Studies with continuous outcomes that required standard deviations not reported were disqualified from the meta-analysis. Due to the absence of pre–post correlation values from the included trials, the post-treatment SMD effect size was utilised. To account for limited sample sizes, Hedges g effect sizes were utilised together with their respective 95% CIs and were interpreted per suggestions. We performed separate meta-analyses for the subdomain group, and we reviewed the secondary/additional outcomes such as adverse effects. Egger’s test and funnel plots were performed to consider publication bias. To determine whether the pooled estimates were consistent, a sensitivity analysis was performed. If the level of heterogeneity was considerable, a meta-regression was attempted. The data were analysed by Review Manager Software (5.4.1 version; The Cochrane Collaboration, Oxford, UK).

## 3. Results

### 3.1. Literature Review of the Randomized Controlled Trials Based on PRISMA Guideline

**Figure 1:**
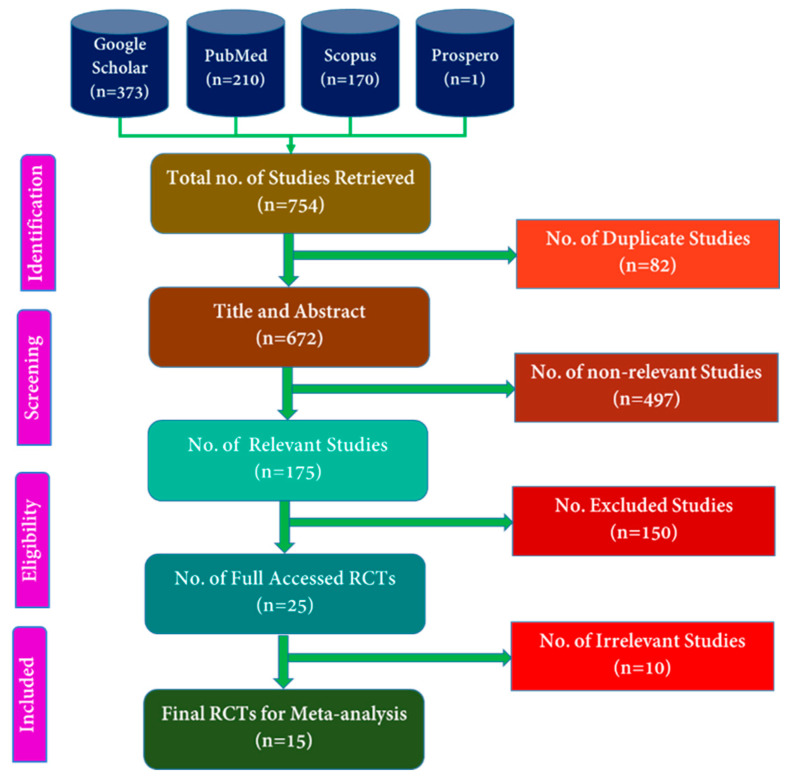
PRISMA flow chart of included publications.

The online search retrieved 754 titles and abstracts of which 82 were duplicates (Figure 1) by using Rayyan online software (https://www.rayyan.ai, accessed on 3 August 2022).

Among the 672 articles, 175 articles were related to PMS, and 497 others were irrelevant. Then, 175 related articles were screened, and 25 full-length RCTs were assessed for eligibility, which were further reviewed for the extraction phase in which 15 full-length RCTs were included for systematic review and meta-analyses that met our inclusion.

We excluded the following RCTs from the meta-analysis as reasoned: Arabnezhad et al. (2022) [1] included a PSST study only before the intervention. Maskani et al. (2020) [31] and Ozgoli (2011) [32] were written in Persian Language, and the data for symptoms were presented as percentages and number. Zamani et al. (2012) [33] used a VAS score for the symptoms, and only a few symptoms were included. Retallick-Brown et al. (2020) [34] in their study on vitamin B6 and micronutrients for PMS used the DSRP tool, and the data were presented in a graph, as well as effect size estimate and 95% confidence intervals. Jafari et al. (2020) [35], in their study on zinc supplements in physical and psychological symptoms before intervention, used the DSM-VI scale; however, pre and post-intervention data for BDNF, serum zinc, hs-CRP, TAC and the average score for physical and psychological factors were calculated. Winther et al. (2018) [36], in their study, only imputed data of VAS score on irritability as a predominant PMS symptom. Delaram et al. (2011) [37], in their study on fennel in PMS, included the daily record of severity of the problem questionnaire (DRSP-Q). Canning et al. (2010) [17], in their study, included total daily symptom report scores as the primary outcome; however, the data were presented as graphs for DSRP. Gerhardsen et al. (2008) [38] studied that women with PMS and included a premenstrual sleep disturbance scale. Agha-Hosseini et al. (2008) [39] investigated *Crocus sativus* L. in PMS and premenstrual daily symptoms (DPS) and the Hamilton depression rating scale (HAMS); however, the data were presented in graphs. Bahrami et al. (2020) [40], in their study on curcumin in PMS, used a premenstrual symptoms scale; however, full length was not available. Esmailpour’s (2019) [41] study used a daily symptoms diary; however, post-intervention data were imputed for nutritional supplements. Ghanbari (2009) [42] studied individual symptoms, and mean and standard deviation were imputed, and a total score was not available.

### 3.2. Characteristics of the Included RCT Studies and Patients

A total of 15 RCTs with 1211 patients, (*n* = 605 and *n* = 606 in intervention and control groups, respectively) with mean ± SD of 80.73 ± 20.39 and CI of 69.44: 92.0 were included in this meta-analysis. Among fifteen studies on PMS, nine studies used PSST scores, two studies PMTS, and the remaining four studies used DSM scoring. Additionally, we carried out PSST scores subgroup analysis for each domain including physical, mood and behavioural symptoms. We found three RCTs on nutritional supplements and twelve RCTs on herbal medicines.

PRISMA methods only covered the given keywords in the Google Scholar, PubMed, Prospero, and Scopus databases for RCT studies. The features of previously published papers included were author name, study design, intervention group, control group, the total number of participants, participants in intervention, control participants, age limit in years, tools, route of administration, duration of intervention, results and side effects/adverse effects of nutritional supplements and herbal medicine, as summarized in Table 1. The age considered was reproductive age. The route of administration included in this study was oral.

### 3.3. Risk of Bias Assessment of the Data

**Figure 2:**
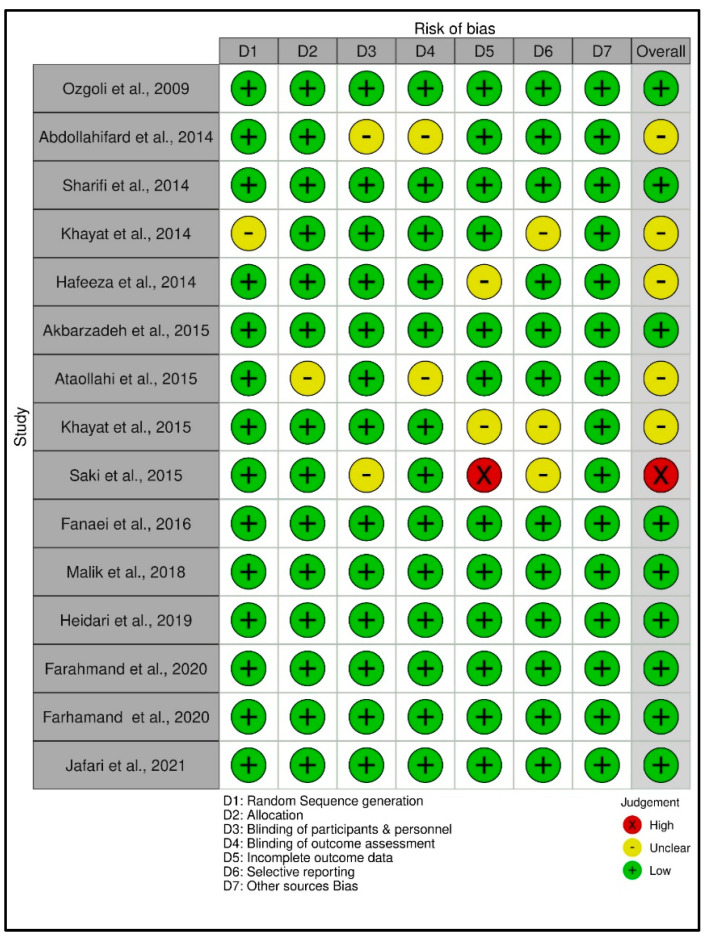
Traffic light plot of randomized controlled trials showing the risk of bias assessment [5,7,43,44,45,46,47,48,49,50,51,52,53,54,55].

**Figure 3:**
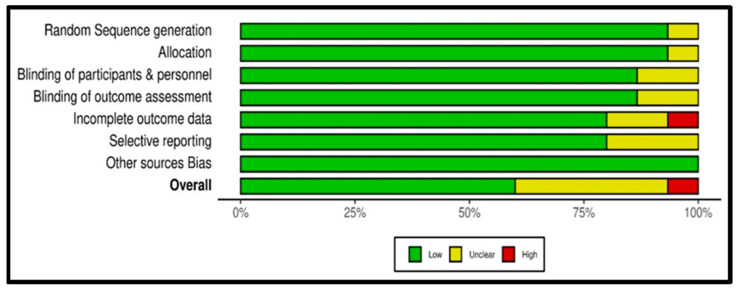
Summary plot of RCTs showing risk of bias.

The risk of bias assessment of the data was depicted through the online software Robvis (https://mcguinlu.shinyapps.io/robvis/, accessed on 12 August 2022) in a generic database, which is a web app designed for visualizing risk-of-bias assessment. Figure 2 (traffic light plot) and Figure 3 (summary plot) summarize the risk of bias assessment of the data of the 15 included studies as per methodological quality using a modified Cochrane Risk of Bias tool for RCTs. 

Bias was measured based on high, low, or unclear individual elements for each domain using a modified Cochrane Risk of Bias tool for RCTs. The red colour in the traffic light plot shows high risk, green colour shows low risk and yellow colour shows unclear risk of bias. The studies of Ozgoli et al. [7], Sharifi et al. [44], Akbarzadeh et al. [47], Fanaei et al. [51], Malik et al. [52], Heidari et al. [53], Farahmand et al. [54], Farahmand et al. [55], and Jafari et al. [5] all had overall low risk of bias, whereas Saki et al. [50] showed an overall high risk of bias (see Figure 2). The studies of Abdollahifard et al. (2014) [43], Khayat et al. (2014) [45], Hafeeza et al. (2014) [46], Ataollahi et al. (2015) [48], and Khayat et al. (2015) [49], showed an overall risk of bias assessment that was unclear. Further, it was observed that studies conducted from 2016 to 2021 showed a low risk of bias.

The quality of included studies was high. All the trials were randomized, three were single-blind, ten studies were double-blind, and two were triple-blind. Out of 15 studies, 4 studies did not report adverse events/side effects, and 11 studies reported adverse events/side effects.

The systematic review showed that 53% of studies (*n* = 8) had a low risk of bias with high quality. One hundred per cent of the studies were randomized. The appropriate method of randomization was reported by 93.33% of studies, while 86.67% reported participant blinding, personnel and blinding outcome assessment. Outcome data incompleteness and selective reporting were reported by 80%, and 100% of studies reported other sources of bias (see Figure 3).

### 3.4. Efficacy of Nutritional Supplements and Herbal Medicine on PMS

#### 3.4.1. Primary Parameters

##### Meta-Analysis for PSST Scores

For PSST scores, we included nine studies. The effect estimate was calculated using standardized mean difference with Hedges g and an applied random effect model with inverse variance. The estimation method applied was restricted maximum likelihood (REML) with no adjustment for standard error. Heterogeneity was considered by the Q test and I^2^. In the study, Q = 18.81 (df = 6, *p* = 0.004), which specifies that the true effect size is similar in all the trials. The Q statistic has poor power to detect actual heterogeneity when a small number of studies are included in the meta-analysis. When many studies are included, it has excessive power to discover trivial variability to then circumvent the limitations of the Q test. The I^2^ statistic is 64% in our study, which shows that variance in the observed effects imitates variance of the true effects to a certain extent versus sampling error. In our analysis, we could not observe significant evidence of high heterogeneity. The Q statistics showed *p* = 0.005; however, in only nine studies, the power to detect heterogeneity was negligible. The variance in the true effect was 0.11. We also employed the Eggers test to determine the degree of funnel plot asymmetry. We found that, in addition to the larger deviation in the intercept, a significant amount of asymmetry was present, as indicated by a *p* value of 0.982. It is also true that this test has poor power, especially when there are fewer than 10 studies and a minimal amount of asymmetry.

**Figure 4:**
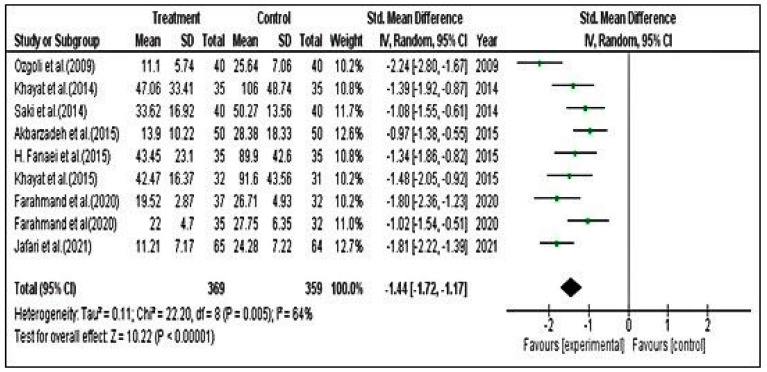
Forest plot for premenstrual screening tool (PSST) [5,7,45,47,49,50,51,54,55].

**Figure 5:**
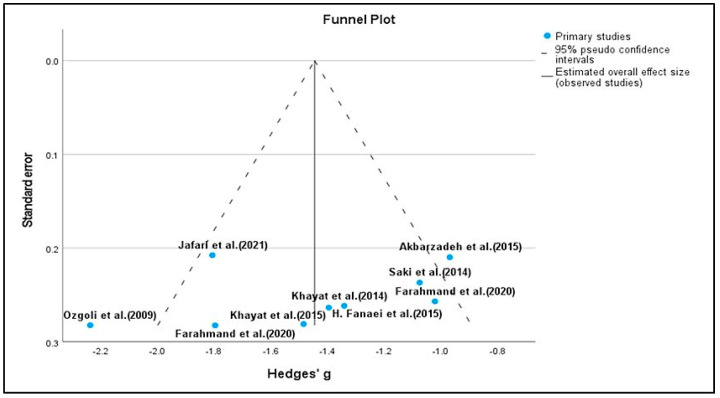
Funnel plot for PSST [5,7,45,47,49,50,51,54,55].

The SMD was −1.44. On average, herbal treatments (intervention group) decreased the PSST score for premenstrual symptoms by −1.44 compared with the control group. The CI for the SMD was [−1.72–−1.17]. This range does not contain an effect size; hence, the mean effect size was not zero. Likewise, the z value for testing the null hypothesis was 10.22, with a corresponding *p* value of <0.0001. Therefore, we conclude that (on average) the herbal drug decreased the PSST score in the population and rejected the null hypothesis, and the true effect size will drop in this range. The prediction interval suggests that in 95% of populations, it is comparable to those in the analysis (Figure 4 and Figure 5).

##### Meta-Analysis for DSR Scores

**Figure 6:**
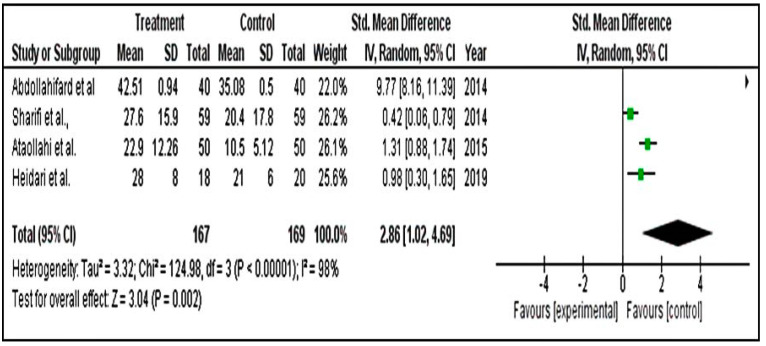
Forest plot for premenstrual screening tool using DSR [43,44,48,53].

For DSR analysis, we included only four studies. The standardized mean difference obtained was 2.86. The overall effect of 2.86 was seen on average compared with the control group. The confidence interval was from 1.02 to 4.69. This range tells us that the mean effect size is not zero. The z value for testing the null hypothesis for effect size, d = 0, is 3.04, with a corresponding *p* = 0.002. Hence, we conclude that herbal drugs decreased the DSR score in the population. Therefore, the impact of the treatment on the DSR score for PMS may not be valid in the population at large. The prediction interval is wide because the number of studies is small (Figure 6).

##### Meta-Analysis for PMTS Scores

**Figure 7:**
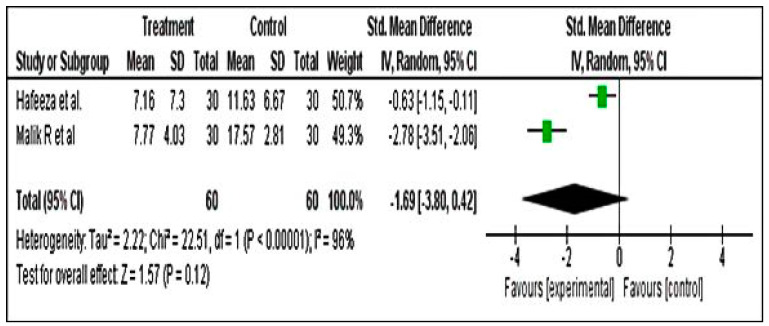
Forest plot for premenstrual screening tool using PMTS [46,52].

The PMTS analysis was carried out including two studies only. The results revealed an overall effect estimate of −1.69 (−3.80–−0.42). The overall effect showed a z value of 1.57 with corresponding *p* = 0.12 (Figure 7).

#### 3.4.2. Secondary Parameters

**Figure 8:**
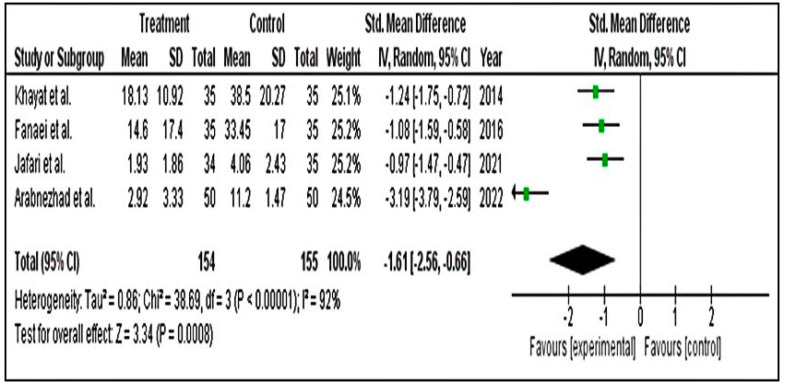
Forest plot for subgroup physical symptoms of PSST scale [1,5,45,51].

**Figure 9:**
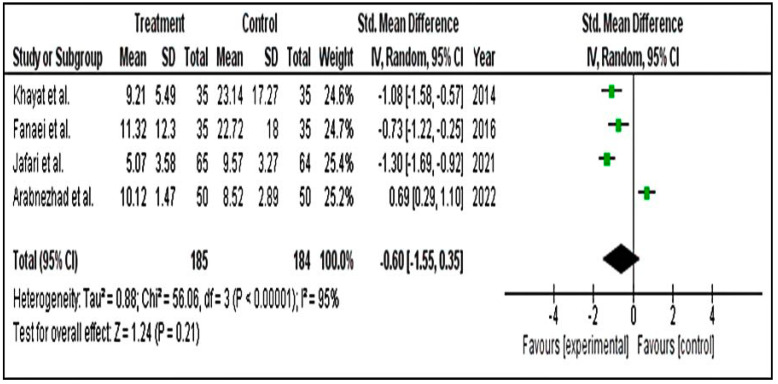
Forest plot for subgroup behavioural symptoms of PSST scale [1,5,49,51].

**Figure 10:**
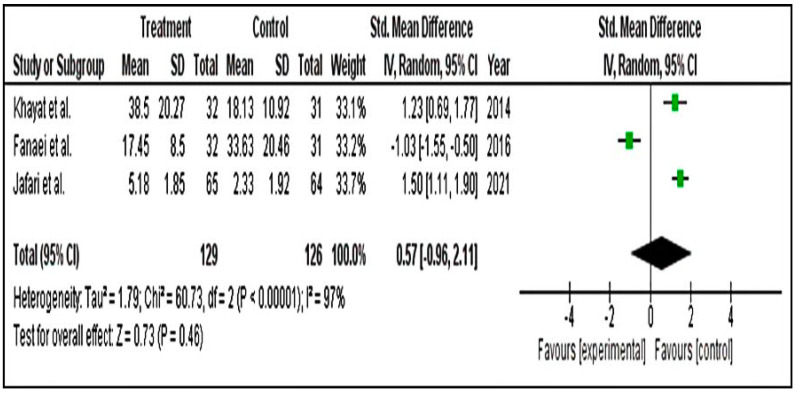
Forest plot for subgroup mood symptoms of PSST scale [5,45,51].

We ran a subgroup group analysis based on the results of physical, behavioural and mood symptoms. Secondary results for physical (SMD = −1.61; 95% CI = −2.56 to −0.66) (Figure 8), behavioural (SMD = −0.60; 95% CI = −1.55 to 0.35) (Figure 9) and mood (SMD = −0.59; 95% CI = −0.96 to 2.11) (Figure 10) symptom groupings of PSST displayed similar findings. From the above finding, we can conclude that, overall, there is a decrease in the symptoms score in the intervention groups. Although the effect size is small, the results are statistically significant.

### 3.5. Safety of Nutritional Supplements and Herbal Medicine

The meta-analysis of the studies showed that nutritional supplements and herbal medicine are relatively safe for PMS. In 15 studies, 11 studies mentioned side effects. Abdollahifard et al. (2014) [43], Ataollahi et al. (2015) [48], Heidari et al. (2019) [53], Farahmand et al. (2020) [54], and Farahmand et al. (2021) [55] reported no side effects (Table 1). However, Ozgoli et al. (2009) [7] reported that *G. biloba* showed mild side effects such as nausea and excessive sleep. Only one patient complained of nausea, and two participants reported an increased desire for sleep, and in the placebo group, four participants reported nausea. Khayat et al. (2014) [45] observed that the ginger group had the only side effect of nausea in one patient. Sharifi et al. (2014) [44] reported that the chamomile group had side effects such as an increase in menstrual bleeding and more severe gastrointestinal problems in the mefenamic group with no other side effects in both groups. Jafari et al. (2021) [5] reported mild effects in the garlic group such as itching, acne and flushing.

### 3.6. Synthesis and Analysis of Previous Studies Related to Herbal Medicine and Nutritional Supplements Related to Premenstrual Syndrome

We designed a database based on previously published studies. It is divided into two parts; (i) Based on Web of Science (WoS) and Scopus websites; (ii) Based on network visualization, and word cloud. The WoS and Scopus are more popular and are authentic indexing websites for research work. Additionally, network visualization and word cloud are more popular now because they are based on software and are easy to implement.

**Figure 11:**
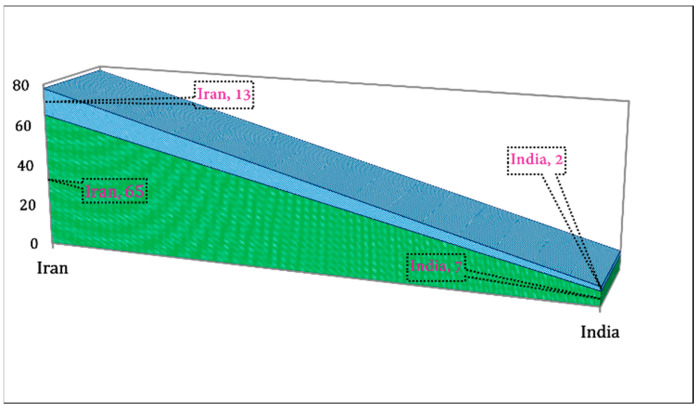
Country-wise and author-wise publications related to RCTs on nutritional supplements and herbal medicine in PMS.

**Figure 12:**
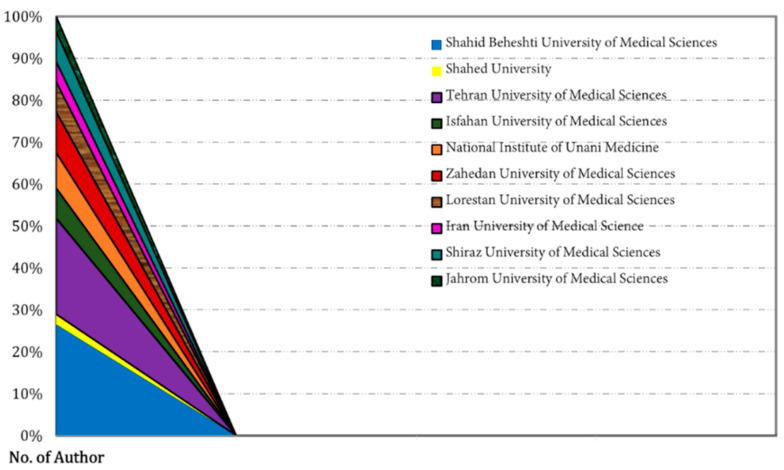
Author distribution as per university-wise related to published RCTs on nutritional supplements and herbal medicine in PMS.

**Figure 13:**
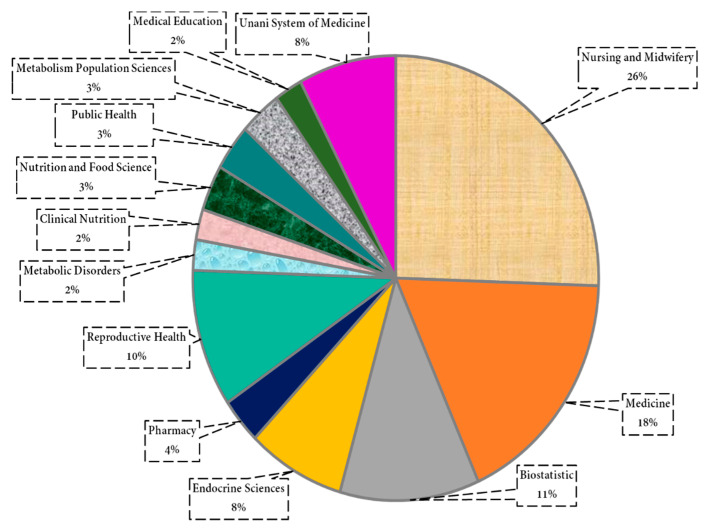
Research area-wise authors in a publication on nutritional supplements and herbal medicine in PMS.

We extracted the data related to the country-wise research area and university/institutions from WoS and Scopus. We found that the Iranian and Indian researchers only worked in this area (Figure 11). The Iranian researcher’s share was found to be 13 out of 15 publications. Additionally, in the university/institution, the highest number of researchers were from Shahid Beheshti University of Medical Sciences (~28%), and the lowest were from Shahed University (~2%) (Figure 12). In the research area, the highest number of researchers were from Nursing and Midwifery (26%) and the lowest from Metabolic Disorders (2%), Clinical Nutrition (2%), and Medical Education (2%), as shown in Figure 13.

**Figure 14:**
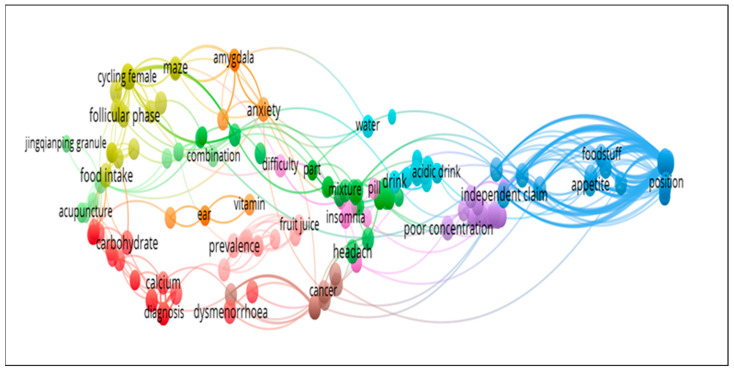
Network visualization based on previously published studies for the collection of the closest terms.

**Figure 15:**
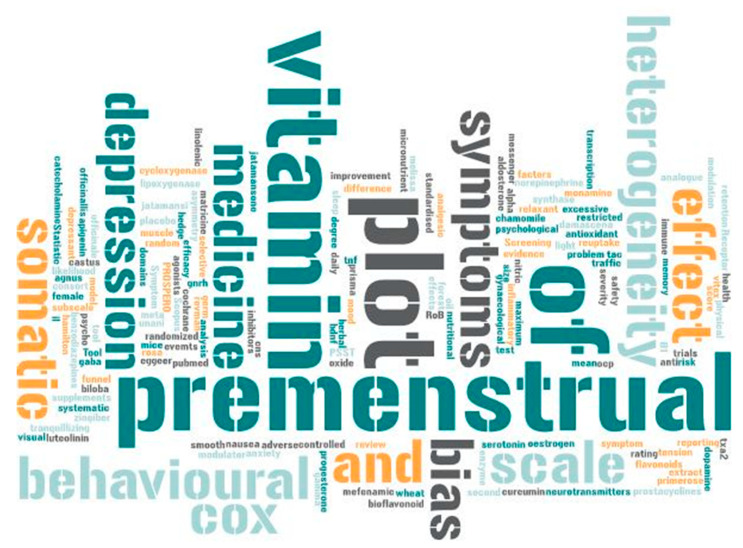
Word clouds of the present study for the collection of the closest terms.

We designed the network visualization [56] based on published article keywords using *VOSviewer* [56], as mentioned in Figure 14. Previously, Heyat’s group used network visualization in the area related to oxidative stress [13], sleep disorders [24,25], motor imagery, education [57], machine learning [58], cryptocurrency [59], and smartphone addiction [26] to write the review. In addition, word clouds based on the present study are mentioned in Figure 15. Heyat’s group also used a word cloud for anxiety [23], stress [58], inflammation [13], camphor [12], deep learning [60], augmented reality [57], and blockchain technology [59] to write the review. These techniques are beneficial to obtain the exact keywords related to any field of research. It is a new tool to analyse previously published publications. In addition, manually, it is difficult to find the closest terms for any big database. These tools will open a new way to visualize the data using *VOSviewer* software. They easily divide the previously published articles based on terms using the cluster.

Our database would be helpful to researchers, doctors, scholars, and scientists to find the exact data based on keywords. It will also open a new way of research and attract new researchers to work in this area, because we have found a very limited number of researchers working in this area.

## 4. Discussion

### 4.1. Major Findings

Nutritional supplements and herbal medicines are used for various medicinal approaches. We explored whether nutritional supplements and herbal medicines would be efficacious and safe for the management of premenstrual somatic and psycho-behavioural symptoms. To the best of our knowledge, there has been no meta-analysis previously conducted on premenstrual somatic and psycho-behavioural symptoms with herbal medicine and nutritional supplements. Our results revealed that there was a significant reduction in the PSST, PMTS, and DSRP scores in the intervention group as the comparator. Furthermore, it was observed that physical, behavioural and mood symptom scores improved significantly in the intervention group. Included trials for the present studies were precisely selected. All studies were randomized controlled trials. All studies had a sample size of 30 and above and were well designed according to the CONSORT guidelines. Tolerable side effects related to nutritional supplements and herbs were reported in four trials. In addition, broadly, we emphasized the low risk and high quality of 15 RCTs in premenstrual somatic and psycho-behavioural symptoms. Additionally, the mechanisms of action of plant metabolites and their bioactive molecules effective in PMS symptoms were also comprehensively discussed.

### 4.2. Comparison with Previously Published Articles

The data of this review inclusively agreed with the published systematic review. However, a meta-analysis of single herbs was available, but none of the studies presented a meta-analysis of nutritional supplements and herbal medicines; they rather wrote the results narratively. The systematic review scrutinized 15 trials that including herbal medicine and nutritional supplements, and it had more participants compared to previous reviews; nonetheless, apprehensions about trial quality remain, along with inadequate reporting details.

Previously, systematic reviews were conducted on PMS regarding herbal medicine and acupuncture (Dante), including 10 RCTs. A systematic study was conducted on the single herb chamomile [6]. Pearce et al. (2020) [8] conducted a systematic review and meta-analysis on exercises for PMS. Other authors included 17 studies on *Vitex agnus castus* [16]. Another study explored dietary supplements and herbal medicines for PMS [17]. One more systematic review on *R. damascena* explored the efficacy of anxiety, fatigue, menstruation-related pain, bloating and headache [18]. Csupor et al. [19], for the management of PMS, compared the efficacy of BNO 1095 and Ze 440 to placebo. The researchers systematically reviewed and included 18 RCTs of Iranian herbal medicines and their effectiveness and safety for PMS [20].

Maleki-Saghooni et al. [20] in their systematic review assessed Iranian herbs for premenstrual syndrome treatment and demonstrated their efficacy and safety. The results of our meta-analysis also shed light. Even in our study, we found that herbal medicine and nutritional supplements can be alternative treatments for PMS symptoms. Verkaik et al. [16] showed that the pooled effect of *Vitex agnus castus* (VAC) was large (Hedges’ g: −1.21; 95% CI: −1.53 to −0.88), but heterogeneity was extremely high. In our study, we found Hedges’ g: −1.44; 95% CI: −1.72 to −1.17. The results of the effect size estimates were slightly higher in our study, and heterogeneity was less (69% vs. 91%). The study also highlighted the need for high-quality trials of suitable size to investigate the effect of vitex in comparison to placebo. In our study, the heterogeneity was comparatively less because we selected good quality studies and based the analysis on a specific scoring system for PSST.

A review by Dante and Facchinett [15] also shows that some herb remedies seem beneficial for PMS treatment. Even our study results are in line with the same. Finally, despite the widespread use of herbs in everyday life, very scarce studies have been devoted to specific clinical investigations. The evidence is scant, but it appears that the relief of only a few herbs could cause relief of PMS. Various studies on herbs showed beneficial effects in decreasing symptoms. Trials should be addressed as comprehensively and thoroughly as possible while adhering to the CONSORT guidelines.

### 4.3. Mechanism of Action of Plant Products

The precise mechanisms for PMS symptoms are uncertain; we hypothesized that oxidative stress probably affects intricate psycho-somatic disease processes such as PMS. Oestrogen and progesterone have antioxidant effects on healthy women, while in women with PMS, pro-oxidant activity is increased, which leads to oxidative damage [61]. Few clinical trials have confirmed that progesterone and allopregnanolone levels are related to depression in women during the premenstrual phase [62]. Still, oxidative stress and chronic inflammation are potential causes of PMS development and other gynaecological diseases. Few studies have measured the biomarkers of oxidative stress and inflammation in PMS symptoms. In addition, allopregnanolone production is altered in PMS, and its blockage ameliorates serotonin re-uptake inhibitors that also affect allopregnanolone levels [13,61]. Furthermore, psycho-somatic PMS symptoms also involve the aetiology of chronic inflammation, and higher correlations were noted for interleukins (12, 10), including their symptoms [53]. Johnson et al. [63] testified that the strongest association was between IL-10 and IL-12 levels and symptoms in PMS women. In mild to moderate premenstrual symptoms, the treatment modality is diet and lifestyle changes, which potentially is a cure. However, if the symptoms create an adversarial effect on daily life, non-pharmacological and pharmacological treatments are suggested, with the last option being surgery.

Plant products include a variety of inorganic and organic constituents (resins, flavonoids, steroids, tannins, and protein) that are proven to have smooth muscle relaxant, tranquillizing, dopamine CNS depressant, analgesic, immune-modulator, antioxidant, and anti-inflammatory effects [64,65,66,67].

Flavonoids have been proven for a higher antioxidant capacity and for scavenging free radicals [68]. They also display anti-inflammatory activity and modulate signal transduction for the synthesis of pro-inflammatory cytokines [14]. They inhibit the synthesis and release of inducible NO synthase, inflammatory IL-6, TNF-a, and MCP-1 by inhibiting NF-κB, AP-1, and other pro-inflammatory transcription factors. Flavonoids suppress the formation of inflammation mediators (arachidonic acid, leukotrienes, and prostaglandins) through second messenger modulation, and they inhibit COX and lipoxygenase activity and arachidonic acid-metabolizing enzymes [14]. Tannins also have antioxidant properties, and therefore scavenge free radicals and stop oxidative damage [64].

Ginkgo improves mood and behavioural symptoms (depression) since it inhibits MOA and TXA2 methyl esterase and increases catecholamine’s neurotransmitters and decreases the re-uptake of these molecules. It also increases blood circulation by maintaining the balance of prostacyclin. Its bioflavonoids have anti-inflammatory activity, as they inhibit cyclooxygenase and lipoxygenase (important for the production of inflammatory prostaglandins) and are stress modulators. In addition, the quercetin bioactive molecule present in Ginkgo is a potent inhibitor of histamine release. Hence, Ozgoli et al. [7] concluded that Ginkgo was able to reduce the severity of symptoms of this mechanism. The active ingredients in Ginkgo are bioflavonoids. Bioactive molecules Gingerol and shogaol, present in *Z. officinalis*, have gained significance, as they possess sedative, anti-inflammatory, and analgesic activities. Ginger decreases platelet aggregation and hence has antimigraine potential. It also acts as an effective PG inhibitor and enhances the release of substance P from trigeminal fibres [69]. Vitamin B1 is a coenzyme that reduces symptoms of PMS by affecting the metabolism of lipids, carbohydrates, and protein. Vitamin B1 may increase endorphin secretion, affect brain and liver function, and release ATP, thereby causing relaxation and stimulating sleep. In addition, it can possibly promote cardiovascular system function. Vit. B1 affects the urinary tract and neurological changes. Hence, relieving PMS symptoms, especially mental symptoms, is reasonable. Vitamin B1 also stimulates the CNS and elevates mood, and subsequently, causes deep relaxation [43]. The active bioactive plant metabolite, a flavonoid present in chamomile, stimulates CNS. The bioactive molecules, Apigenin and Luteolin, act as antianxiety and relief as their bind with benzodiazepine receptors [44]. Chamazulene present in chamomile extract has been found accountable for anti-inflammatory activity. Matricine and (-)-alpha-bisabolol also show anti-inflammatory and analgesic activities. (-)-alpha-bisabolol, spiroethers and apigenin exhibit a spasmolytic effect [69]. *Vitex agnus castus* has dopaminergic compounds, which are clinically important and improve premenstrual mastodynia and other PMS symptoms [69]. The bioactive molecule Jatamansone found in *N. jatamansi* in experimental animals (mice and monkeys) exerted a tranquillizing effect. In rabbits, jatamansone reduced 5HT in the brain, as it impairs the biosynthesis of serotonin, although the degradation of serotonin was unaffected. Hence, the mode of action of jatamansone was thus in variance with that of reserpine, which has a direct action on the cell to liberate serotonin. Contrary, the roots of Indian Nard’s alcoholic extract cause upsurges in central monamines levels, 5-hydroxyindole acetic acid and inhibitory amino acids, GABA, dopamine, norepinephrine, and serotonin in rat brains [69]. The bioactive molecule found in anise seed, anethole, has a structure similar to catecholamines, including noradrenaline, adrenaline, and dopamine [69]. GABA is an inhibitory neurotransmitter, acting as a neuronal suppressor. It is released from vesicles and activates the GABA receptor family in the postsynaptic membrane. It has been assumed that GABAergic deficits are related to the occurrence of depression. Earlier studies have shown that GABA concentrations in the occipital and prefrontal cortex are knowingly lower in patients with depression [62].

Micronutrients also enhance QoL, and they have been beneficial to reduce PMS symptoms; nevertheless, studies of their efficacy are scarce. Zinc is an essential micronutrient required in human metabolism and is known to play antioxidant and anti-inflammatory roles in the human body [70]. Some studies support that vitamin intake is effective in reducing PMS symptoms. Arabnezhad et al. [1] reported that curcumin improves serum Vit D levels and shows a remarkable increase in serum TAC levels, reproducing an augmentation in antioxidant status in PMS women [53]. Various research studies have confirmed that flowers have gamma-linolenic acid (GLA), which has antioxidant and anti-inflammatory properties and is beneficial to the severity and duration of PMS symptoms. Evening primrose oil also contains GLA and is beneficial in relieving PMS symptom severity at 4 to 6 months follow-up after treatment [71]. In addition, PMS may cause reductions in antioxidant activity and increases in oxidative stress [72].

Various scientific studies have recognised that curcumin can control neurotransmitters (norepinephrine, dopamine, serotonin, and BDNF) accountable for behaviour and mood regulation [51,73]. Chen et al. [62] found that BDNF may be involved in the neuro steroids-mediated regulation of the hypothalamic–pituitary–adrenal (HPA) axis. A study [49] reported that curcumin inhibits the COX-2 enzyme, relieves physical symptoms during menstruation, and improves mood and behavioural symptoms of PMS. In stressed animals, curcumin exhibited strong antidepressant effects, similar to SSRIs (fluoxetine and imipramine), and prevents a decrease in BDNF levels of the hippocampus [51]. Numerous studies have established that phytoestrogens bind to oestrogen receptors and show significant oestrogenic-like effects [74]. The anise seed mechanism of action is similar to SERMs and has agonistic and antagonistic effects on the estrogenic receptors. Anethole acts as the active oestrogenic agent present in anise seed [55].

### 4.4. Strength of the Study

This updated study has numerous strengths. PRISMA guidelines were used. The PROSPERO-P guideline was followed to write the protocol and was registered in PROSPERO. A complete search approach, established in coordination with comparable reviews, was performed, and electronic databases and grey literature were explored. We performed the determined risk of bias assessment using the Cochrane tool for systematic reviews. For a systematic review of the present study, we also designed a database for country, number of authors, and university/institution from previously published publications. We included RCTs in this review and meta-analysis, and it is anticipated as the highest quality of evidence presently accessible. For the first time, this systematic review and meta-analysis quantitatively summarize the accessible evidence on the objectives of whether nutritional supplements and herbal medicine are effective treatments of premenstrual somatic and psycho-behavioural symptoms. In addition, side effect reporting included in the studies was discussed. Furthermore, we substantially added artificial intelligence including word clouds and network visualization based on keywords.

Nevertheless, this systematic review and meta-analysis are dissimilar to other systematic reviews, as very few publications have included both systematic reviews and meta-analyses. In addition, we summarized the mechanism of action of plant metabolites and their bioactive molecules in herbal medicines. Furthermore, we used SPSS v.28.0.0 (IBM, Armonk, NY, USA) and Rayyan software and Cochrane risk assessment to analyse the risk of assessment of randomized controlled trials. Network visualization was also performed for retrieval of articles from the Web of Science. This study will not only help academicians but also health care professionals and scientists to work in this field and use AI for data recovery from numerous databases. Additionally, our nearby terms might be supportive for academicians, students, researchers, and doctors to find the closest articles concerned with our study.

### 4.5. Research Gaps, Implications, and Practices

There are some potential research gaps in the study. Moderate to high heterogeneity was observed. However, with only nine studies of PSST, the power to detect heterogeneity was negligible. One of the main reasons for high heterogeneity was the inclusion of studies with various types of herbal formulations. We included nutrition studies as well. The asymmetry of the funnel plot may indicate publication bias, however, due to the limited number of trials. There is a considerable likelihood that deviations from the optimum funnel shape could be the result of chance. Furthermore, a skewed funnel plot can also be brought on by elements other than publication bias, such as the calibre of the study, the variation in intervention intensity, the variation in underlying risk, the choice of the effect measure, and chance. Finally, according to BMJ guidelines, when there are less than 10 papers included in the meta-analysis, testing for funnel plot asymmetry should generally not be utilized because the test power is typically insufficient to distinguish between chance and actual asymmetry [75]. Nevertheless, this meta-analysis can be seen as a first step in providing a formal examination of the hypothesis. The effect size values for PSST and the prediction interval reveal that there will be some populations where the impact of the treatment is large, and somewhere it is trivial. We could not generate evidence which proves that there could be a large effect or impact on the population as a whole. However, it shows relatedly better results in the improvement of various symptoms as compared to the control group.

This systematic review and meta-analysis were conducted through four electronic databases with restrictions to the English language that probably affect the results. In addition, there are a low number of studies for each included study per outcome that perhaps preclude us from assessing publication bias, and the results are relatively suggestive versus conclusive, for which larger RCTs are recommended to study in the future. We would also like to caution the readers that some of the included studies have a high risk of bias based on the Cochrane quality assessment tool for randomized trials. We found that the most recent studies (58%) were low risk. However, future RCTs should stand by methodological standards to reduce bias as much as practicable.

In this paper, the research gap is obvious and consequently should necessitate further investigation. In addition, future trials should plan time concerning the CONSORT guidelines to improve the trial’s quality. Furthermore, larger-scale studies with a large sample size among females from various backgrounds and community socioeconomic levels, utilizing various herbal medicine dosages for longer periods, are recommended. Some studies that do not use a placebo suggested achieving more definitive results concerning the effectiveness and safety of herbal medicine for PMS relief symptoms. Certainly, additional research is crucial to investigate the connection between a given mechanism of action of herbs and nutritional supplements in clinical trials, although limited animal studies have proven anti-inflammatory, sedative, dopamine, antidepressant, SSRIs and antioxidant properties of herbs and nutritional supplements at the molecular level. Hence, additional investigation is needed. Researchers should develop more precise scales to outline PMS. Furthermore, emerging technologies, such as block chain technology, computer vision, quantum techniques, machine learning, and brain network can help to analyse experimental RCT data.

This updated systematic review and meta-analysis supports the suggestion that physicians and healthcare workers caring for PMS women may consider the use of nutritional supplements and herbal medicine as part of their care plan. This may potentially help PMS symptoms and do so with carefulness until good quality evidence is available. The research recommendations are focused to refine writing practices and procedures to enhance the quality of forthcoming trials. It is recommended that forthcoming research in this field must use either the PSST or the DSR alone or both and report global symptom changes such as those reported here. Additional information related to the perseverance of the positive effects of nutritional supplements and herbal medicines beyond the end of the trial period could be studied. If the sample size is large, then subgroup analysis by contraceptive use, age, or other demographic factors might be performed by further systematic reviewers.

## 5. Conclusions

This updated systematic review and meta-analysis can be a breakthrough towards investigating nutritional supplements and herbal medicines that are possibly effective natural substitutes to relieve premenstrual somatic and psycho-behavioural symptoms. However, there is to date inadequate evidence to conclude whether nutritional supplements and herbal medicine can treat PMS. In addition, we concluded that network visualization and word cloud techniques are easy to use to design a comprehensive diagram of any study. This diagram would be more helpful to upcoming researchers to work in this area and to easily collect exact data. Further study should be designed for the meta-analysis and systematic review of clinical trials of other female disorders.

## Figures and Tables

**Table 1:** The features of the published RCTs on nutritional supplements and herbal medicines.

Authors	Study Design	Interven.	Control	Part.	Age (y)	Tools	Route of Admin., Durat. and Dosage	Durat. of Interven. (Cycles)	Result	Adv. Event	Ref.
Ozgoli et al. (2009)	Single blind	*Gingko biloba* L. tablet	Placebo	90	18–30	PSST	One tablet (containing 40 mg leaf extracts) three times per day from the 16th day of the menstrual cycle to the 5th day of the next cycle	2	Severity of symptoms reduced significantly	Reported (Nausea and excessive sleep in intervention group)	[7]
Abdollahifard et al. (2014)	Double Blind	B1 (Thiamine)	Placebo (Starch powder)	80	18–30	DSR	Two pills of Vit B1 (each pill contains 100 mg) twice daily	3	Reduces mental and physical symptoms	Reported (No side effects)	[43]
Sharifi et al. (2014)	Double blind	*M. chamomile* extract	Mefenamic acid 250 mg TID	90	18–35	DSR	100 mg capsules thrice daily from the 21st day until the next onset of menstruation period, three times daily for two cycles	2	Chamomile is more effective in relieving symptoms	Reported (Excessive bleeding in intervention group and GI complication in MA group)	[44]
Khayat et al. (2014)	Double blind	*Z. officinale* capsules	Placebo	70	18–35	PSST	Two capsules 250 mg/12 h (7 days) before menstruation to three days after menstruation	3	Reduction in mood, physical, and behavioural symptoms	Reported (Complaint of nausea in the intervention group)	[45]
Hafeeza et al. (2014)	Single blind	*V. agnus castus* seed and *Mentha piperita* Linndistillate (*Arq Pudina*) 72 mL	Placebo	60	13–40	PMTS-SR, PMTS-O	*V. agnus castus* seed 1 g and *M. piperita* distillate 36 mL were administered orally twice daily, 10 days before menstruation in every cycle	3	Significant reduction in PMTS score in the intervention group	Not reported	[46]
Akbarzadeh et al. (2015)	Double blind	*Melissa. officinalis* Linn (*Badranjboya*) essence capsules	Placebo (starch)	100	-	PSST	2 capsules (1200 mg) daily from the first to the last day of their menstrualcycle	3	Effective in reduction of symptoms	Not reported	[47]
Ataollahi et al. (2015)	Triple blind	*Triticum aestivum* Linn (Wheat germ) extract	Placebo	100	20–45	DSR	I capsule (400 mg), three times per day between the 16th day of the menstrual cycle to the 5th day of the next menstrual period	2	Wheat germ significantly reduced physical (63.56%), psychological (66.30%), and the general score (64.99%	Reported (No side effects)	[48]
Khayat et al. (2015)	Double Blind	Curcumin from *Curcuma longa* Linn (haldi)	Placebo (brown sugar)	70	-	PSST	Two capsules (100 mg) BID daily for seven days before menstruation and three days after menstruation	3	Reduction in symptoms	Reported (No side effects)	[49]
Saki et al. (2015)	Triple blind	*Oenothera biennis* *Linn* (Primrose) oil	Placebo (*n* = 40)	80	18–30	PSST	3 capsules (1500 mg) TID per day	3	Significant relief in symptoms	Not reported	[50]
Fanaei et al. (2016)	Double blind	Curcumin capsules	Placebo (Brown sugar)	70	-	DSR Fasting Serum BDNF level	1 capsule of 100 mg/12 h was given for 10 days (in each menstrual cycle 7 days before and 3 days after onset of menstrual bleeding)	3	Significant relief in symptoms and increased level of BDNF in the intervention group	Not reported	[51]
Malik et al. (2018)	Single blind	*Nardostachys jatamansi* (D. Don) DC. (*jatamansi*) capsules	Placebo (Roasted wheat flour)	60	18–45	PMTS-O, PMTS-SR	3 capsules orally, BD for the 15 days before the expected date of menstruation, up until the onset of the next menstrual cycle	2	PTMS and VAS scores were significantly reduced in the intervention group	Reported (No side effects)	[52]
Heidari et al. (2019)	Double blind	50,000 IU of vitamin D3	Placebo pearl fortnightly	44	18–25	PMS Daily Symptoms Rating form	50,000 IU of vitamin D3 for fortnightly	4	Significant improvement in 25(OH) D, serum IL-12, and TAC levels.	Reported (No side effects)	[53]
Farahmand et al. (2020)	Double blind	Flowers of *Echium* *amoenum* Fisch. & C A Mey (*Gole gauzaban*)	Placebo	84	20–35	PSST	Capsules 450 mg of TID from the 21st day to the 3rd day of their next cycle	2	Improve PMS symptoms	Reported (No side effects)	[54]
Farahmand et al. (2021)	Double blind	*P. anisum* seed	Placebo (starch)	84	18–35	PSST	110 mg capsules of Anise three times per day started 7 days before the start of the menstruation and continued until 3 days after menses	2	Significant relief of symptoms	Reported (No side effects)	[55]
Jafari et al. (2021)	Double blind	*Allium* *sativum* Linn (lahsun) tablet (1.1 mg allicin)	Placebo (Starch tablet)	129	15–49	PSST	One tablet (400 mg) daily	3	Significant reduction in symptoms	Reported (Mild complaints)	[5]

DSR: Daily Symptom Report; DRSP-Q: Daily Record of Severity of Problem Questionnaire; PMTS-O: Premenstrual Tension Observer Questionnaire; PMTS-SR: Premenstrual Tension Self-Rating Questionnaire; PSST: Premenstrual Symptoms Screening Tool Questionnaire.

## Data Availability

Not applicable.
